# Detecting electrophysiological alterations in psychiatric disorders through event-related microstates: a systematic review

**DOI:** 10.1192/j.eurpsy.2025.815

**Published:** 2025-08-26

**Authors:** F. F. Marzocchi, A. Perrottelli, G. Di Lorenzo, C. D’Amelio, E. Caporusso, G. M. Giordano, L. Giuliani, A. Melillo, P. Pezzella, P. Bucci, A. Mucci, S. Galderisi

**Affiliations:** 1 University of Campania “Luigi Vanvitelli”, Naples; 2 University of Rome “Tor Vergata”, Rome; 3 University of L’Aquila, L’Aquila, Italy

## Abstract

**Introduction:**

Measurements of event-related potentials (ERP), recorded through electrophysiology (EEG) during sensory and cognitive processing tasks, have been widely employed to investigate the pathophysiological basis of mental health disorders. In the last two decades, the analysis of EEG microstates (MS) has been increasingly applied to ERP data. This methodology allows the detection of spatio-temporal changes in neuronal activity potential at the scalp level, providing new insights into neurobiological alterations detectable in psychiatric disorders.

**Objectives:**

The current systematic review aims at providing an extensive and detailed description of the studies that characterized alterations in ERP-microstates in psychiatric and neurodevelopmental disorders.

**Methods:**

A systematic review of English articles using PubMed, Scopus and Web of Science databases was undertaken in April 2024. The review included case-control studies that employed ERP-microstates analysis to compare MS features between subjects (age > 10 years) diagnosed with a mental health disorder and healthy controls.

**Results:**

Out of the 756 records screened, 15 studies were included in the final qualitative synthesis. The studies included patients with schizophrenia (n=7), alcohol use disorder (n=2), borderline personality disorder (n=1), panic disorder (n=1), autism (n=1), major depressive disorder (n=1), post-traumatic stress disorder (n=1) and attention-deficit/hyperactivity disorder (n=1).

The studies investigated alterations of MS characteristics through different types of tasks. Cartool and RAGU were the main software used for MS analysis. Only rarely studies used similar tasks, showing comparable microstate maps. Fourteen of 15 studies showed a significant difference (p<0.05) in MS characteristics between patients and healthy controls. Main differences regarded parameters such as duration, area under the curve and the order of occurrence of MS.

**Conclusions:**

The present literature review aims to highlight the effectiveness in using microstate analysis to identify spatio-temporal alterations in brain electrical activity in subjects with psychiatric disorders, showing the possible implications of the use of this technique in clinical practice and its advantages, as compared to ERP peak analysis.

**Disclosure of Interest:**

None Declared

